# Untreated for 20 Years: A 14 Kilogram Subcutaneous Lipoma

**DOI:** 10.29252/wjps.7.3.368.

**Published:** 2018-09

**Authors:** Joseph A. Ricci

**Affiliations:** The Brigham and Women’s Hospital, Department of Surgery, Division of Plastic and Reconstructive Surgery, Harvard Medical School, Boston, MA, USA

**Keywords:** Giant lipoma, Multi-disciplanary approach, Reconstructive surgery, Liposarcoma, Teratoma

## Abstract

Lipomas are the most common benign mesenchymal tumors found in humans, with a prevalance rate of 2.1 per 1000 tumors. Most of them are small, weighing only a few grams and measuring less than 2 centimeters in diameter. However, those weighing upwards of 200 grams and exceeding 10 centimeters have only been described in different anatomic locations on occasion in the literature. A 54-year-old man presented with a large soft tissue growth over the lower back, present for the past 20 years and rapidly enlarging over the past 3 years. A diagnosis of giant lipoma was made. Six hours and several surgical teams were required to remove the 14 kilogram mass. During excision, the skin flaps overlying the mass were preserved and were ultimately used to reconstruct the surgical site defect in a layered fashion once intraoperative frozen pathology confirmed the pre-operative diagnosis. Benign lipomas of the size reported in this case have rarely been described in the literature, as lipomatous masses of this size are often found to instead be either atypical lipomatous tumors or high-grade liposarcomas. In this case, we describe one of the largest of these giant benign lipomas ever reported to date. The case also illustrates the use of an interdisciplanary, multi-team approach to undertake the high-risk operation of removing such a mass from a patient safely. Finally, the case describes aninteresting approach toward reconstructing the large soft tissue defect that remained once the tumor had been removed from the patient.

## INTRODUCTION

The largest reported cutaneous lipoma to date was 22.7 kilograms and was removed by Brandler off the left shoulder of a 26-year-old patient in 1894.^1^^,^^2^ Lipomas occur with an estimated prevalance rate of 2.1 per 1000 tumors and are among the most common benign mesencymal tumors found in humans.^2^^,^^3^ Most lipomas are small, often measuring less than two centimeters in diameter and weighing only a few grams.^2^ On occasion, those weighing upwards of 200 grams and exceeding 10 centimeters have been described in the literature in different anatomic locations.^2^^-^^6^ The rarity of benign lipomas of this size has led patients with similar large soft tissue masses to rather be evaluated for sarcomas or teratomas.^5^

In the case of giant benign lipomas, a complete resection is often possible. However, given the size of these lesions, the resultant soft tissue defecit can often be quite large and as a result can be challenging for plastic surgeons to reconstruct. In this manuscript, we report the case of one of the largest benign lipomas ever recorded in the literature. The case is not reported beause of the rarity of the condition, since lipomas are one of the most common benign masses, but because of itslarge size. The enormity of the mass highlights some of the unique challenges associated with the diagnosis, surgical resection and reconstruction of these giant benign tumors.

## CASE REPORT

**Fig. 1:**
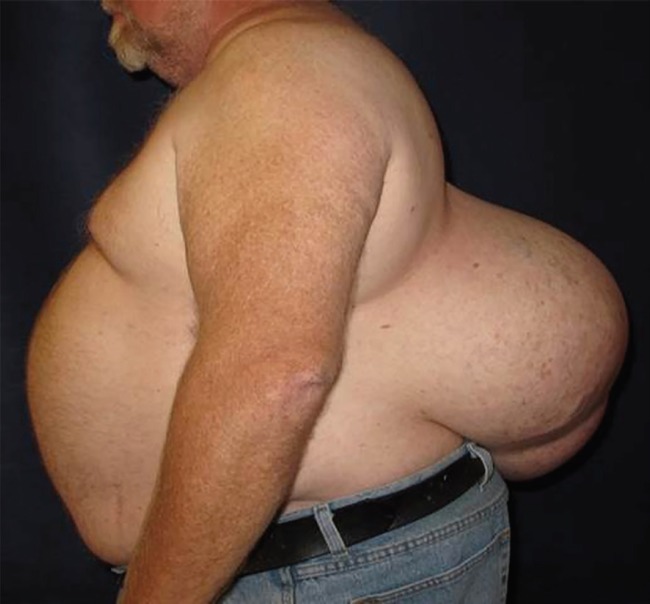
Pre-operative photo of the patient in the lateral view

A 54-year-old man with a past medical history significant only for hypertension presented to our clinic with a large soft tissue growth on hislower back which had been present for the past 20 years. Over the past three years it had been rapidly enlarging, nearly doubling in size over that time frame. He had recently re-established medical care after having not seen a physician since childhood. At the time of presentation, he denied any pain or tenderness over the mass and denied any systemic symptoms such as fever, night sweats, and weight loss. The patient had an unremarkable physical exam except for the large soft tissue mass over the lower back, with the maxiumum dimension measured to be 38cm (Figure 1) .

After evaluation by the surgical oncology and radiation oncology services, an abdominal CT scan was obtained and thisdemonstrated a large (35 cm, x 38 cm x 17 cm), heterogeneous soft tissue mass. A differential diagnosis consisting of teratoma versus liposarcoma was established based on the radiologic imaging. Subsequently, several core biopsies of the mass were performed, all of which revealed fat necrosis with calcifications.Surprisingly, given the size and rapid growth of the mass, a diagnosis of benign giant lipoma was made. 

**Fig. 2:**
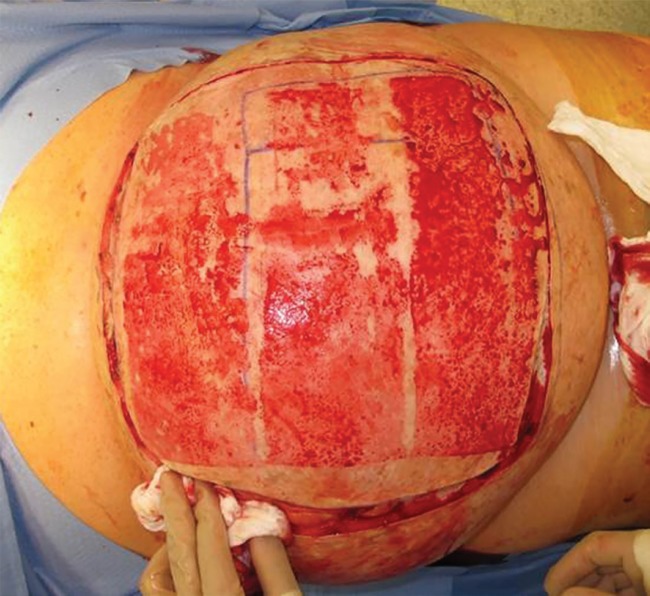
Intra-operative photo demonstrating the harvest of skin grafts and initial skin- preserving incision. The patient’s head is to the left

**Fig. 3:**
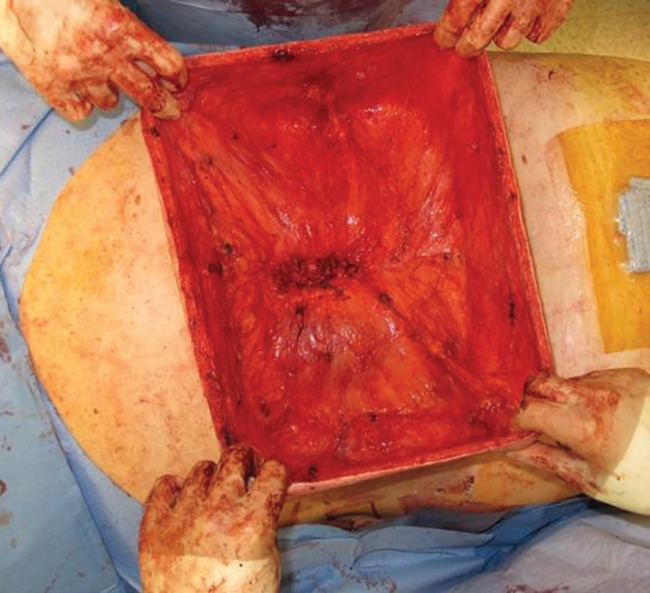
Intra-operative photo of the remaining skin flaps used for reconstruction after tumor enucleation prior to de-epithelialization and imbrication. The patient’s head is to the left

Four weeks after presentation, several surrgical teams performed a six hour operation to remove the 14 kilogram mass. After the patient was widely prepped and draped, the skin overlying the central portion of the tumor was shaved and harvested as multiple split thickess skin grafts (Figure 2). Subsequently, an incision was made in the skin overlying the tumor in an area outside the skin graft donor sites, preserving significant flaps in all dimensions to permit primary closure (Figure 3). Numerous, large variceal vessels feeding the tumor were ligated as the tumor was dissected off of the paraspinous muscles, which constituted the deep margin.

**Fig. 4:**
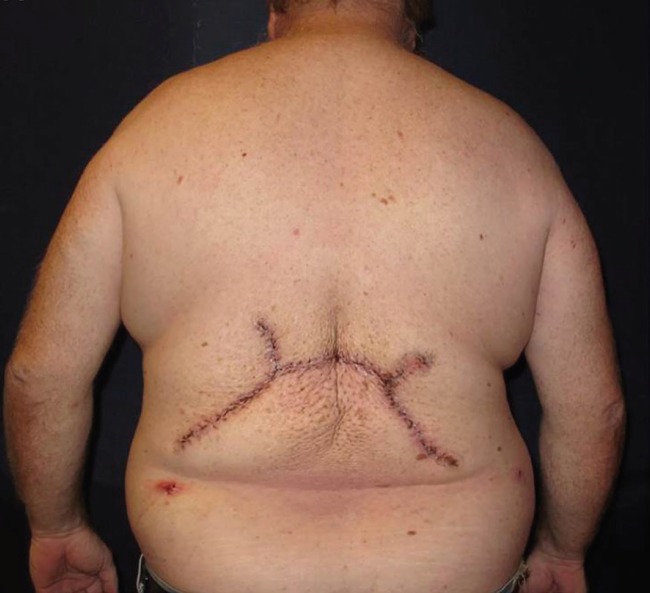
Post-operative photo of the patient in the posterior view, demonstrating the final closure

The specimen was sent for frozen section analysis,which was consistent with a lipoma, and was confirmed on final the pathology. The defect was able to be closed primarily with the preserved skin flaps, which measured greater than 200 cm × 40 cm (Figure 3). The skin flaps were de-epithelialized and imbricated to achieve a multi-layered closure of the entire back wound, obliterating as much of the deadspace as possible. Two subcutaneous closed-suction drains were placed prior to the final closure. Postoperatively, the patient did well without complication (Figure 4). After a brief and uneventful hospital stay postoperatively, he was discharged home in good condition. On follow-up, his drains were sequentially removed and the incision line has healed without problems. He has not had any evidence of recurrence or infection at six months postoperatively.

## DISCUSSION

The patient in this case had not accessed the health care system for over 20 years and initially presented to a new primary care physician for evaluation of this mass. The initial treating physician promptly referred the patient to our institution given the very uncommon situation of a patient presenting with a mass of this size. As in this case, the identification and treatment of such a large mass is best performed at a tertiary care center where a patient can be evaluated by a multidisciplanary team of physicians with extensive experience in managing large soft tissue tumors.^4^^,^^6^

The plastic surgeons have an important role as part of this team, as they are often required to assist with the closure of the large and often complex soft tissue defects that result after resection of such a mass. This can often be a daunting task considering patients can receive radiation therapy to the area and are sometimes malnourished. For these reasons, it is critical for the plastic surgeon to evaluate these patients pre-operatively and be an integral part of the pre-operative planning from the outset.^4^^,^^6^

At our center, the longstanding relationship we have with the surgical oncologists enabled this patient to not only receive a prompt diagnosis but also allowed his wound to be completely reconstructed without the need for local muscle flaps, skin grafting or free tissue transfer. Using a combined multi-team approach, the skin envelope surrounding this large mass was able to be preserved during the resection so that it could be used to achieve primary wound closure at the end of the case. Additionally, although we did not ultimately use them, split-thickness skin grafts were harvested from the skin overlying the tumor at the begnining of the case with the plan to use them for reconstruction in the event that the preserved skin flaps might prove insufficient or unsuitable for wound closure due to ischemia or positive resection margins.^4^^,^^6^

The ability to perform this patient’s reconstruction was a direct result of the collaboration between both the plastic surgeon and surgical oncologist in the operating room for the duration of the case as well as the meticulous pre-operative planning by an interdisciplanary team of phycsicians. As illustrated by this report, we advocate that plastic surgeons actively participate as part of pre- and intra-operative interdisciplanary teams when undertaking a complex operation to remove such a mass from a patient in the safest manner possiblein order to achieve the best possible outcome.

## CONFLICT OF INTEREST

The authors declare no conflict of interest.
